# Supplementary material for: Content-rich biological network constructed by mining PubMed abstracts
Source: BMC Bioinformatics. 2004 Oct 8;5:147. doi: 10.1186/1471-2105-5-147 (PMC528731; doi:10.1186/1471-2105-5-147)
Supplement: Additional File 2 — The original results of the above study (non-essential files are deleted to keep the file size under the limit set by BMC bioinformatics). [file 1471-2105-5-147-S2.bz2 › chilibotAdditionalFile2/dip20/212ID7768894E718/html/CSNK2B_CSNK2A2.html]

 


 **CSNK2B** and **CSNK2A2** 
  
Found 141 abstracts in PubMed, retrieved 20.  
 

 What does Google say? 
 PDF only 
| .edu only 

---

**Interactive relationship** (e.g. stimulation, inhibition, etc)

**Neutral relationship**- Phosphorylation of EYFP 4E BP1 by the mitogen activated protein kinase ERK2, but not by casein kinase CK II  [ **CSNK2A2** ]   [ **CSNK2B** ] , also attenuated the interaction.  Ref: 11404183 Int J Biochem Cell Biol, 2001
- We found that the stress induced kinase casein kinase CK II  [ **CSNK2A2** ]   [ **CSNK2B** ]  phosphorylates the Ser276 immediately preceding the tyrosine motif, increasing AQP4 mu 3A interaction and enhancing AQP4 lysosomal targeting and degradation.  Ref: 11742978 EMBO J, 2001

**Non-interactive relationship** (e.g. studied together, co-existance, homology, etc.)

- Rhodnius CK II  [ **CSNK2A2** ]   [ **CSNK2B** ]  is strongly inhibited by heparin Ki = 9 nM and uses ATP Km = 36 microM or GTP Km = 86 microM as phosphate donors.  Ref: 12110292 Insect Biochem Mol Biol, 2002
- In addition, the phosphorylation of the recombinant N in vitro can be blocked by a CK II  [ **CSNK2A2** ]   [ **CSNK2B** ]  inhibitor, heparin.  Ref: 12711319 Biochem Biophys Res Commun, 2003
- ii purified both proteins function as phosphate acceptors of CK II  [ **CSNK2A2** ]   [ **CSNK2B** ] .  Ref: 11041245 Biol Pharm Bull, 2000
- The recombinant N was phosphorylated by BHK cellular extracts and by purified CK II  [ **CSNK2A2** ]   [ **CSNK2B** ] .  Ref: 12711319 Biochem Biophys Res Commun, 2003
- LFs to a glycyrrhizin GL affinity column and their phosphorylation by casein kinase II CK II  [ **CSNK2A2** ]   [ **CSNK2B** ]  in vitro were biochemically investigated.  Ref: 11041245 Biol Pharm Bull, 2000
- Thus, our data demonstrate that cellular CK II  [ **CSNK2A2** ]   [ **CSNK2B** ]  phosphorylates rabies virus N.  Ref: 12711319 Biochem Biophys Res Commun, 2003
- and iii oGA at one tenth the concentration of GL inhibits the CK II  [ **CSNK2A2** ]   [ **CSNK2B** ]  mediated stimulation of sPLA2 IIA activity.  Ref: 11558558 Biol Pharm Bull, 2001
- We have obtained a 3000 fold VT free enriched preparation of CK II  [ **CSNK2A2** ]   [ **CSNK2B** ] .  Ref: 12110292 Insect Biochem Mol Biol, 2002
- 6.6 nM, which is about 30 fold lower than that observed with CK II  [ **CSNK2A2** ]   [ **CSNK2B** ]  beta.  Ref: 11226880 J Biochem Tokyo , 2001
- Characterization of a CK II  [ **CSNK2A2** ]   [ **CSNK2B** ]  protein kinase from etiolated oat seedlings.  Ref: 12243354 Mol Cells, 2002
- Enhanced casein kinase II CK II  [ **CSNK2A2** ]   [ **CSNK2B** ]  activity in human lung tumours.  Ref: 12017291 Anticancer Res< MedlineTA>Anticancer Res, 2002
- The physiological correlation between glycyrrhizin GL and high mobility group proteins I and 2 HMG1 2 and the inhibitory effect of GL on their phosphorylation by three protein kinases CK I, CK II  [ **CSNK2A2** ]   [ **CSNK2B** ]  and PKC were investigated biochemically in vitro.  Ref: 11510483 Biol Pharm Bull, 2001
- These properties identify the major protein kinase activity as a type II casein kinase CK II  [ **CSNK2A2** ]   [ **CSNK2B** ] .  Ref: 11446384 Insect Biochem Mol Biol, 1993
- Both purified HMG1 2 functioned as phosphate acceptors for these two protein kinases CK I and PKC, but not phosphorylated by CK II  [ **CSNK2A2** ]   [ **CSNK2B** ] .  Ref: 11510483 Biol Pharm Bull, 2001
- Furthermore, it was found that i purified sPLA2 IIA is phosphorylated by casein kinase II CK II  [ **CSNK2A2** ]   [ **CSNK2B** ]  in vitro.  Ref: 11558558 Biol Pharm Bull, 2001
- and ii CK II  [ **CSNK2A2** ]   [ **CSNK2B** ]  mediates stimulation of its PLA2 activity in vitro.  Ref: 11558558 Biol Pharm Bull, 2001
- Characterization of secretory type IIA phospholipase A2 sPLA2 IIA as a glycyrrhizin GL binding protein and the GL induced inhibition of the CK II  [ **CSNK2A2** ]   [ **CSNK2B** ]  mediated stimulation of sPLA2 IIA activity in vitro.  Ref: 11558558 Biol Pharm Bull, 2001
- MATERIALS AND METHODS The aim of the present study was to evaluate the activity of CK II  [ **CSNK2A2** ]   [ **CSNK2B** ]  both in neoplastic and non neoplastic human lung tissue by using a synthetic peptide with Arg Arg Arg Asp Asp Asp Ser Asp Asp Asp RRRDDDSDDD sequence.  Ref: 12017291 Anticancer Res< MedlineTA>Anticancer Res, 2001
- These results suggest that the GL induced inhibition of the DNA binding affinity and the CK II  [ **CSNK2A2** ]   [ **CSNK2B** ]  mediated phosphorylation of hLF may be closely correlated with the anti inflammatory effect of GL in the human body.  Ref: 11041245 Biol Pharm Bull, 2000
- hondai on the activities of four distinct Ser Thr protein kinases two casein kinases CK I and CK II  [ **CSNK2A2** ]   [ **CSNK2B** ] , A kinase and C kinase were determined in vitro.  Ref: 12612451 Biol Pharm Bull, 2003
- Using the multifunctional protein kinase CK II  [ **CSNK2A2** ]   [ **CSNK2B** ] , we have shown that incorporation of phosphate into P1A protein can exert its effect on the hetero oligomerization process, namely by preventing the formation of the hetero oligomer P1A P P2B.  Ref: 11118639 Biochim Biophys Acta, 2000
- These results, therefore, indicate that the purified kinase is a CK II  [ **CSNK2A2** ]   [ **CSNK2B** ]  protein kinase and may be involved in the regulation of DNA topoisomerase I activity.  Ref: 12243354 Mol Cells, 2002
- CONCLUSION Our results suggest that CK II  [ **CSNK2A2** ]   [ **CSNK2B** ]  plays an important role in cellular proliferation and the mechanism of its activation.  Ref: 12017291 Anticancer Res< MedlineTA>Anticancer Res, 2002
- In this study we show that Vitellin VT phosphorylation in chorionated oocytes of Rhodnius prolixus is completely inhibited by heparin 10 microg ml, a classical casein kinase II CK II  [ **CSNK2A2** ]   [ **CSNK2B** ]  inhibitor.  Ref: 12110292 Insect Biochem Mol Biol, 2002
- 0.25 microM and casein kinase II CK II  [ **CSNK2A2** ]   [ **CSNK2B** ] , ID 50 = approx.  Ref: 11226880 J Biochem Tokyo , 2001
- However, no significant effect of the CK II  [ **CSNK2A2** ]   [ **CSNK2B** ]  mediated hLF phosphorylation on its DNA binding affinity was detected.  Ref: 11041245 Biol Pharm Bull, 2000
- The reading frame encodes a polypeptide consisting of 526 amino acid residues, containing a number of DNA binding motifs and phosphorylation sites for PKC, CK II  [ **CSNK2A2** ]   [ **CSNK2B** ] , and p34cdc2.  Ref: 12736779 J Mol Med< MedlineTA>J Mol Med, 2000
- One unit of CK II  [ **CSNK2A2** ]   [ **CSNK2B** ]  activity is defined as the amount catalysing the transfer of 1 pmol 32P into the substrate within 1 minute at 37 degrees C.  Ref: 12017291 Anticancer Res< MedlineTA>Anticancer Res, 2000
- The activity range of CK II  [ **CSNK2A2** ]   [ **CSNK2B** ]  was between 500 1500 U mg protein.  Ref: 12017291 Anticancer Res< MedlineTA>Anticancer Res, 2000
- Previous studies indicated that inositol hexasulphate inhibited the activity of CK I and or CK II  [ **CSNK2A2** ]   [ **CSNK2B** ]  in mouse tooth germs Torres Quintana et al.  Ref: 11077997 J Dent Res, 2000
- Phosphorylation by protein kinase C PK C or casein kinase II CK II  [ **CSNK2A2** ]   [ **CSNK2B** ]  within or near the nonhelical tail end domain inhibits assembly of MIIB alpha F47 Murakami, N.  Ref: 10985790 Biochemistry, 2000
- Incubation of VT with purified Rhodnius CK II  [ **CSNK2A2** ]   [ **CSNK2B** ]  and 32 P ATP led to the incorporation of 2 mols of phosphate mol VT.  Ref: 12110292 Insect Biochem Mol Biol, 2002
- BACKGROUND Casein kinase II CK II  [ **CSNK2A2** ]   [ **CSNK2B** ]  is expressed at a higher level in lung tumours when compared to the corresponding non neoplastic lung tissue.  Ref: 12017291 Anticancer Res< MedlineTA>Anticancer Res, 2002
- Physical, biochemical and antigenic properties of this kinase identify it as casein kinase II CK II  [ **CSNK2A2** ]   [ **CSNK2B** ] .  Ref: 11861906 Nucleic Acids Res, 2002
- Replacement of the CK II  [ **CSNK2A2** ]   [ **CSNK2B** ]  or PK C sites with Asp MIIB alpha F47 CK 5D or PK 4D strongly inhibited the filament assembly, with or without Mg 2, by significantly increasing the critical concentrations for assembly.  Ref: 10985790 Biochemistry, 2000
- The respective serines are located in conserved recognition sites for luminal protein kinases protein kinase CK II  [ **CSNK2A2** ]   [ **CSNK2B** ]  and Golgi casein kinase.  Ref: 12033762 Virology, 2002
- Furthermore, N phosphorylation in the virus infected cells can be inhibited by a CK II  [ **CSNK2A2** ]   [ **CSNK2B** ]  specific inhibitor, dichloro beta D ribofuranosyl benzimidazole.  Ref: 12711319 Biochem Biophys Res Commun, 2003
- The monoclonal antibodies CK II  [ **CSNK2A2** ]   [ **CSNK2B** ]  and A45 B B3 were used with the immuno cytochemical standard method for detecting IDT BM.  Ref: 12678519 Int J Surg Investig, 2000
- These data show that an insect yolk protein contain phosphorylation sites for a cyclic nucleotide independent protein kinase such as CK II  [ **CSNK2A2** ]   [ **CSNK2B** ] .  Ref: 12110292 Insect Biochem Mol Biol, 2002
- The same mutations also abolish phosphorylation of eIF5 when transfected into mammalian cells suggesting that CK II  [ **CSNK2A2** ]   [ **CSNK2B** ]  phosphorylates eIF5 at these two serine residues in vivo as well.  Ref: 11861906 Nucleic Acids Res, 2002
- Additionally, we show here that human PHI is phosphorylated at serine 185 by casein kinase II CK II  [ **CSNK2A2** ]   [ **CSNK2B** ]  and we provide experimental evidence suggesting that this phosphorylation is associated with secretion, thus providing insights for elucidating the intracellular signal transmission of cell response to stimulation by AMF NLK MF.  Ref: 11004567 Biochim Biophys Acta, 2000
